# Immune classification for the PD-L1 expression and tumour-infiltrating lymphocytes in colorectal adenocarcinoma

**DOI:** 10.1186/s12885-020-6553-9

**Published:** 2020-01-28

**Authors:** Byeong-Joo Noh, Jae Young Kwak, Dae-Woon Eom

**Affiliations:** 1 Department of Pathology, Gangneung Asan Hospital, University of Ulsan College of Medicine, Gangneung, South Korea; 2 Department of Surgery, Gangneung Asan Hospital, University of Ulsan College of Medicine, Gangneung, South Korea; 3 Department of Pathology, Gangneung Asan Hospital, 38, Bangdong-gil, Sacheon-myeon, Gangneung-si, Gangwon-do 25440 Korea

**Keywords:** PD-L1, CD8, Tumour microenvironment immune type, Colorectal adenocarcinoma

## Abstract

**Background:**

Colorectal adenocarcinoma is the third most common cancer worldwide and a leading cause of cancer-related death. The recent emergence of diverse immunotherapeutic agents has made it crucial to interpret a complex tumour microenvironment intermingled with tumour-infiltrating immune cells to predict the immunotherapeutic response rate. However, in colorectal adenocarcinoma, studies are lacking that provide detailed analyses of programmed death-ligand 1 (PD-L1) and tumour-infiltrating lymphocytes (TIL) to elucidate their prognostic values and to identify immunotherapy-targetable subgroups, preferably with multiple immune-related biomarkers. In the present study, we categorize colorectal adenocarcinomas into four types of tumour immune microenvironments according to PD-L1 expression and TIL, analyse their prognostic values, and propose an immunotherapy-targetable subgroup.

**Methods:**

Formalin-fixed, paraffin-embedded tissue samples of surgically resected primary colorectal adenocarcinomas (*n* = 489) were obtained and arrayed on tissue microarray blocks. Immunohistochemical stains for PD-L1, programmed cell death protein 1 (PD-1), cluster of differentiation 8 (CD8), and deficient mismatch repair (dMMR) were performed and evaluated.

**Results:**

Tumour microenvironment immune type (TMIT) I (PD-L1-positive tumour cells and CD8-high TIL) and type II (PD-L1-negative tumour cells and CD8-low TIL) showed the best and worst prognoses, respectively. PD-L1 overexpression was significantly associated with dMMR status. PD-L1 immunoreactivity was positively correlated with TIL having CD8 or PD-1 overexpression.

**Conclusions:**

TMIT I subgroup showed stronger CD8/PD-L1/PD-1 signalling interaction compared to the other TMIT. Therefore, we propose that the TMIT I subgroup is a candidate TMIT to predict effective response rate for existing immune checkpoint inhibitors and determine targetable subgroups for emerging therapies.

## Introduction

Colorectal adenocarcinoma is the third most common malignancy worldwide and a leading cause of cancer-related mortality, and its occurrence is increasing [1]. Multimodal therapies such as surgery, chemotherapy, and radiotherapy have been the typical first-line therapies for colorectal adenocarcinoma. Recently, the tumour microenvironment has been emphasized, and analyses of the interactive relationships between tumour cells and the immune environment have received much attention. Regulation of the immune system through immune checkpoint inhibitors is an emerging therapeutic approach. Immunotherapies that target programmed cell death-ligand 1 (PD-L1) or programmed cell death-1 (PD-1) inhibitors have become cornerstones of treatments for malignant tumours such as gastrointestinal, pulmonary, renal cell carcinoma, and melanoma [2–5].

In colorectal adenocarcinoma, PD-L1 or PD-1 expression, tumour mutational burden (TMB), tumour-infiltrating lymphocytes (TIL), and microsatellite instability (MSI) have been accepted as fundamental biomarkers that guide the clinical application of immune checkpoint inhibitors based on the following lines of evidence [6]: 1) PD-L1 expression is significantly associated with favourable [7] or unfavourable [8–10] prognostic values except some studies [11, 12], and blocking PD-L1/PD-1 interaction can prolong tumour suppression and stabilize the progression of advanced cancers [13]; 2) the response rate to immune checkpoint inhibitors is significantly associated with increased tumour mutation burden [14]; 3) PD-L1-positive tumour cells and CD8-positive TIL are key prognostic biomarkers for locally advanced rectal cancer patients treated with neoadjuvant chemoradiotherapy [15]; 4) MSI is significantly associated with a prolonged response rates and favourable clinical outcomes in colorectal and non-colorectal cancer patients treated with immune checkpoint inhibitors [16].

Immunotherapeutic agents are recommended to limited subpopulations of patients based on biomarker expression patterns that have been associated with clinical efficacy and response rates. Representative biomarkers for tumour immune microenvironments are required to distinguish responsive patient subgroups and to predict therapeutic outcomes. However, difficult hurdles remain for deciphering tumour immune microenvironments, pioneering representative novel biomarkers, and stratifying immunotherapy-targetable patients because of the multiplicity of immunotherapy agents, tumour heterogeneity, variable immune suppression mechanisms, and the complexity of interactions between tumours and patients’ immune systems [17]. To date, in colorectal adenocarcinoma, selection criteria that can reliably detect specific subgroups of patients whose tumours will respond to available immunotherapies are lacking. Therefore, further elucidation of clinical response in patients whose tumours have specific combinations of representative biomarkers is imperative.

In the present study, we categorized 489 colorectal adenocarcinomas into four tumour immune microenvironment types (TMIT) based on representative biomarkers such as PD-L1 expression and the presence of TIL. We also conducted clinicopathologic and prognostic analyses with each TMIT, and from the results, we propose an immunotherapy-targetable subgroup.

## Materials and methods

### Patients and clinicopathologic data

This study was approved by the Institutional Review Board of Gangneung Asan Hospital. We collected 489 cases of primary colorectal adenocarcinomas originating in the mucosa of the colon and rectum that were surgically resected between 2004 and 2012 in Gangneung Asan Hospital (Gangneung, Republic of Korea). Exclusion criteria were as follows: 1) histological diagnosis of a tumour type other than adenocarcinoma, 2) inappropriate numbers of tumour cells, and 3) insufficient preservation of paraffin blocks for tissue microarray (TMA) construction.

Demographic and clinicopathologic data were collected from patient medical records, including the patient’s gender and age, surgical resection date, most recent follow-up date, and the patient’s local recurrence or survival status. Pathology was assessed using haematoxylin and eosin (H&E)-stained slides. Pathological data included tumour size, location, pTNM stage, the histological subtype, tumour differentiation, lymph node metastasis, and lymph vascular or perineural invasion.

### Tissue microarray

Formalin-fixed, paraffin-embedded (FFPE) tissue samples were selected and arrayed using a TMA instrument (Quick-Ray, Unitma Co., Ltd., Seoul, Korea). Briefly, representative areas of each case were reviewed and marked on the H&E-stained slide, and its corresponding FFPE block was sampled with a 2-mm-diameter tissue cylinder. The sampled tissue was transferred to a recipient block. Four μm-thick sections were prepared from TMA blocks for immunohistochemical staining.

### Immunohistochemistry

**Fig. 1:**
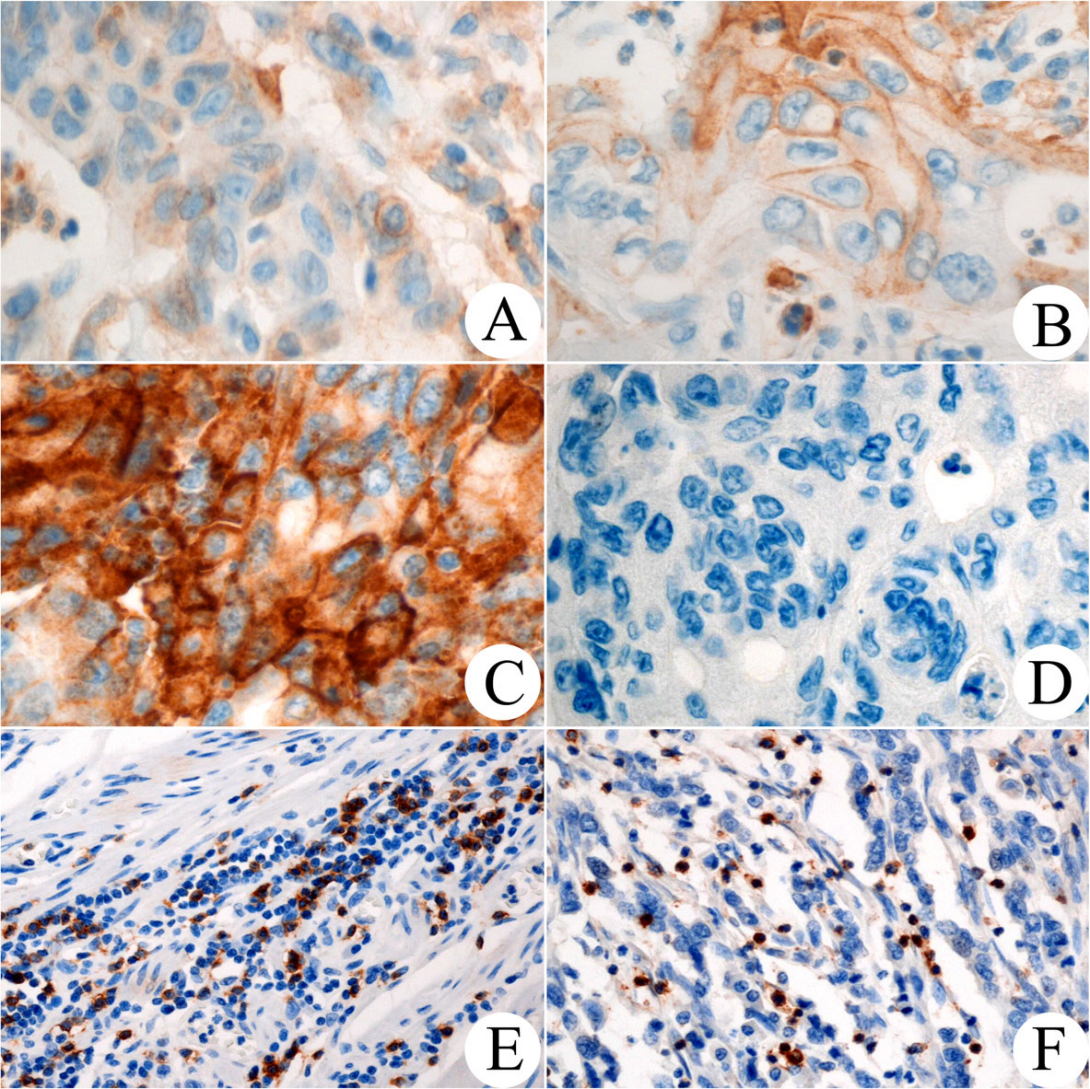
Immunohistochemical staining of PD-L1, CD8, and PD-1. Intensities for positive PD-L1 immunohistochemical stains with a membranous pattern are graded as weak (**a**), moderate (**b**), and strong (**c**), and a negative pattern is shown in (**d**). High expression of CD8 (**e**) and PD-1 (**f**) are also revealed on the tumour bed (magnification: A-F, 400X)

Immunohistochemical (IHC) staining for PD-L1 (SP263; Roche Diagnostics, Tucson, USA; Predilution), PD-1 (EPR4877; Abcam, Cambridge, UK; 1:100), CD8 (SP16; Thermo Fisher Scientific, Runcorn, UK; 1:100), MLH1 (ES05; Leica Biosystems, Newcastle, UK; 1:200), MSH2 (25D12; Leica Biosystems, Newcastle, UK; 1:100), PMS2 (EPR3947; Cell Marque, Hotsprings, USA; predilution), and MSH6 (44; Roche Diagnostics, Tucson, USA; predilution) was conducted using a Bond-Max automated immunostaining device (Leica Biosystems, Newcastle, UK) or a Ventana Benchmark automated staining system (Ventana Medical Systems, Tucson, USA) based on the manufacturer’s recommendations. As positive controls, we used placenta for PD-L1, tonsil for PD-1 and CD8, and colon carcinoma for MLH1, MSH2, PMS2, and MSH6. Negative controls were performed by omitting the primary antibody. Representative stains for PD-L1, CD8, and PD-1 are shown in Fig. 1.

### Immunohistochemical analyses

Immunostaining was assessed blindly by two independent pathologists (BJ NOH and DW EOM). Discrepancies were resolved by simultaneous re-evaluation, and a consensus decision was made.

A semiquantitative assessment for PD-L1 immunoreactivity was obtained by light microscopy. Membranous immunostaining was interpreted based on the proportion and intensity of positive tumour cells. Intensity was graded as 0 (negative), 1 (weak), 2 (moderate), or 3 (strong). The proportion of positive tumour cells was graded as 0 (negative), 1 (< 1%), 2 (1–10%), 3 (11–50%), or 4 (> 50%). Immunoreactive scores (IRS) were calculated by summing these values, culminating in final values ranging from 0 to 7. Positive PD-L1 expression was defined as an IRS value of ≥3.

Immunostaining of PD-1 and CD8 in immune cells was estimated for TIL on the tumour bed area including the tumour epithelium and intratumour stroma by light microscopy (400X; BX51; Olympus, Tokyo, Japan). Five non-contiguous areas including the densest immune cells were selected to ensure that the samples were representative and to increase homogeneity. The numbers of immune cells in the five fields were combined and then averaged to calculate the mean value for one 200X microscopic field (0.1590 mm^2^/field). Mean values (PD-1, 19.0; CD8, 35.0) were utilized as cut-off values to categorize the PD-1 and CD8 expression levels for TIL as “high” or “low”.

A four-tiered classification of TMIT was applied as follows: Type I, positive PD-L1 expression in tumour cells and high CD8 expression in TIL; Type II, negative PD-L1 expression in tumour cells and low CD8 expression in TIL; Type III, positive PD-L1 expression in tumour cells and low CD8 expression in TIL; and Type IV, negative PD-L1 expression in tumour cells and high CD8 expression in TIL. These subgroups have been proposed to determine immunotherapy-targetable patients that are predictive for the best response rates [18].

### Statistical analysis

Statistical analysis was conducted using SPSS software version 23.0 (SPSS Inc., Chicago, IL, USA). Categorical data were analysed with chi-squared or Fisher’s exact tests. Survival curves were illustrated using the Kaplan-Meier method, and log-rank tests were used to calculate relationships between survival rates and various clinicopathologic factors in univariate analyses. We also estimated the prognostic significance using Cox proportional hazards modelling in multivariate analyses. Statistical significance was defined as *p* <  0.05.

## Results

### Clinicopathologic characteristics

Demographics with baseline clinicopathologic characteristics are listed in Table 1. Patients’ ages ranged from 31 to 93 years (mean, 66.6 years; standard deviation (SD), 11.5 years). The male-to-female ratio was 1.51, with a male preponderance. The follow-up period after surgical resection ranged from one day to 164.8 months (mean, 58.3 months; median, 57.0 months).

**Table 1:** Demographics

Parameters	No. of Cases (%)
Age	
Less than 67 yrs	212 (43.4)
More than 67 yrs	277 (56.6)
Sex	
Male	294 (60.1)
Female	195 (39.9)
Size	
Less than 4.9 cm	256 (524)
More than 4.9 cm	233 (47.6)
Location	
Ascending	110 (22.5)
Transverse to sigmoid	242 (49.5)
Rectum	122 (24.9)
NA^a^	15 (3.1)
Histologic type	
Intestinal type	480 (98.2)
Mucinous type	9 (1.8)
Differentiation	
Well	52 (10.6)
Moderately	410 (83.8)
Poorly	18 (3.7)
NA^a^	9 (1.8)
Lymphovascular invasion	
Absence	327 (66.9)
Presence	145 (29.7)
NA^a^	17 (3.5)
Perineural invasion	
Absence	299 (61.1)
Presence	52 (10.6)
NA^a^	138 (28.2)
pT category	
pT1–2	81 (16.6)
pT3	354 (72.4)
pT4	54 (11.0)
Lymph node metastasis	
Absence	276 (56.4)
Presence	213 (43.6)
Stage	
I	65 (13.3)
II	206 (42.1)
III	196 (40.1)
IV	22 (4.5)
Deficient mismatch repair	
Colon	
Absence	335 (91.0)
Presence	33 (9.0)
Rectum	
Absence	113 (93.4)
Presence	8 (6.6)
Chemo-or Radiotherapy	
Only surgical resection	179 (36.6)
Adjuvant chemotherapy	267 (54.6)
Neoadjuvant chemoradiotherapy	14 (2.9)
Adjuvant chemoradiotherapy	29 (5.9)
Local recurrence	
Negative	304 (61.8)
Positive	185 (37.8)
Disease-specific death	
Alive	291 (59.7)
Dead	197 (40.3)
PD-1 expression	
Low	304 (62.2)
High	177 (36.4)
NA^a^	8 (1.6)
CD8 expression	
Low	305 (62.4)
High	173 (35.4)
NA^a^	11 (2.2)
PD-L1 expression	
Negative	302 (61.8)
Positive	179 (36.6)
NA^a^	8 (1.6)
Tumour microenvironment immune type	
Type I (PD-L1+/CD8H)	90 (18.4)
Type IV (PD-L1−/CD8H)	83 (17.0)
Type III (PD-L1+/CD8L)	87 (17.8)
Type II (PD-L1−/CD8L)	218 (44.6)
NA^a^	11 (2.2)

^a^Not assessable due to no clinical information, cautery artifact, fragmentation, or incorrect orientation of tumour tissues

From a total of 489 patients, samples without assessable staining due to cautery artifact, fragmentation, or incorrect orientation of tumour tissues were excluded for PD-L1, PD-1, CD8, and deficient mismatch repair (dMMR). Positive PD-L1, high PD-1, and high CD8 expression were detected in 179 (36.6%), 177 (36.4%), and 173 (35.4%) samples, respectively. We classified each tumour sample into a TMIT according to immunohistochemical results and arranged the TMIT in order of prognostic value (type I, IV, III, and II) as follows: Type I, 90 samples (18.4%); Type IV, 83 (17.0%); Type III, 87 (17.8%); and Type II, 218 (44.6%). dMMR was seen in 41 samples (8.4%) (33 samples (9.0%) in colon; 8 samples (6.6%) in rectum).

### Clinicopathological correlation of TMIT and CD8 expression

Clinicopathological relationships between TMIT and CD8 or PD-L1 expression are delineated in Table 2. TMIT I demonstrates a more significant association with low T category (*p* <  0.001) and decreased lymph node metastasis (*p* = 0.001), culminating in favourable survival (*p* = 0.002) benefit and decreased local recurrence (*p* = 0.031), than the other TMIT. In addition, Type I tended to dMMR (*p* = 0.006).

**Table 2:** Clinicopathologic correlation of tumour microenvironment immune types, CD8 expression, and PD-L1 expression

Parameters		Tumour microenvironment immune type (%)						CD8 expression (%)			PD-L1 expression (%)		
			Type I(PDL1+/CD8H)	Type IV(PD-L1−/CD8H)	Type III(PD-L1+/ CD8L)	Type II (PD-L1−/CD8L)	*P*	Low	High	*P*	Neg.	Pos.	*P*
Age	Less than 67 yrs	36 (17.4)		36 (17.4)	35 (16.9)	100 (48.3)	0.366	135 (65.2)	72 (34.8)	0.575	136 (65.4)	72 (35.6)	0.303
	More than 67 yrs	54 (19.9)		47 (17.3)	52 (19.2)	118 (43.5)		170 (62.7)	101 (37.3)		166 (60.8)	107 (39.2)	
Sex	Male	58 (20.3)		57 (19.9)	46 (16.1)	125 (43.7)	0.094	171 (59.8)	115 (40.2)	0.026*	182 (63.2)	106 (36.8)	0.821
	Female	32 (16.7)		26 (13.5)	41 (21.4)	93 (48.4)		134 (69.8)	58 (30.2)		120 (62.2)	73 (37.8)	
Size	Less than 4.9 cm	50 (20.0)		48 (19.2)	43 (17.2)	109(43.6)	0.227	152 (60.8)	98 (39.2)	0.15 l2	157 (62.5)	94 (37.5)	0.911
	More than 4.9 cm	40 (17.5)		35 (15.4)	44 (19.3)	109 (47.8)		153 (67.1)	75 (32.9)		145 (63.0)	85 (37.0)	
Location	Ascending	26 (24.3)		18 (16.8)	22 (20.6)	41 (38.3)	0.912	63 (58.9)	44 (41.1)	0.849	59 (54.6)	49 (45.4)	0.517
	Transverse to sigmoid	34 (14.2)		41 (17.2)	42 (17.6)	122 (51.0)		164 (68.6)	75 (31.4)		164 (68.3)	76 (31.7)	
	Rectum	27 (23.1)		22 (18.8)	20 (17.1)	48 (41.0)		68 (58.1)	49 (41.9)		70 (59.3)	48 (40.7)	
Histologic type	Intestinal type	89 (18.9)		83 (17.7)	87 (18.5)	211 (44.9)	0.080	298 (63.4)	172 (36.6)	0.160	295 (62.4)	178 (37.6)	0.145
	Mucinous type	1 (12.5)		0 (0.0)	0 (0.0)	7 (87.5)		7 (87.5)	1 (12.5)		7 (87.5)	1 (12.5)	
Differentiation	Well	9 (17.6)		14 (27.5)	8 (15.7)	20 (39.2)	0.795	28 (54.9)	23 (45.1)	0.458	34 (65.4)	18 (34.60	0.483
	Moderately	75 (18.7)		66 (16.5)	76 (19.0)	184 (45.7)		260 (64.8)	141 (35.2)		251 (62.3)	152 (37.7)	
	Poorly	5 (27.8)		3 (16.7)	3 (16.7)	7 (38.9)		10 (55.6)	8 (44.4)		10 (55.6)	8 (44.4)	
Lymphovascular invasion	Absence	65 (20.4)		47 (14.8)	58 (18.2)	148 (46.5)	0.955	206 (64.8)	112 (35.2)	0.503	196 (61.1)	125 (38.9)	0.211
	Presence	21 (14.7)		34 (23.8)	26 (18.2)	62 (43.4)		88 (61.5)	55 (38.5)		96 (67.1)	47 (32.9)	
Perineural invasion	Absence	58 (19.7)		43 (14.6)	59 (20.0)	135 (45.8)	0.650	194 (65.8)	101 (34.2)	0.808	178 (60.3)	117 (39.7)	0.116
	Presence	7 (14.0)		11 (22.0)	7 (14.0)	25 (50.0)		32 (64.0)	18 (36.0)		36 (72.0)	14 (28.0)	
pT category	pT1–2	26 (32.9)		17 (21.5)	15 (19.0)	21(26.6)	< 0.001*	36 (45.6)	43 (54.4)	0.001*	178 (60.3)	117 (39.7)	0.001*
	pT3	58 (16.8)		56 (16.2)	65 (18.8)	166(48.0)		231 (67.0)	114 (33.0)		36 (72.0)	14 (28.0)	
	pT4	6 (11.1)		10 (18.5)	7 (13.0)	31 (57.4)		38 (70.4)	16 (29.6)		178 (60.3)	117 (39.7)	
Lymph node metastasis	Absence	68 (25.3)		43 (16.0)	47 (17.5)	111 (41.3)	0.001*	158 (58.7)	111(41.3)	0.009*	154 (56.8)	117 (43.2)	0.002*
	Presence	22 (10.5)		40 (19.1)	40 (19.1)	107 (51.2)		147 (70.3)	62 (29.3)		148 (70.5)	62 (29.5)	
Stage	I	24 (37.5)		11 (17.2)	11 (17.2)	18 (28.1)	0.001*	29 (45.3)	35 (54.7)	0.006*	29 (45.3)	35 (54.7)	0.003*
	II	41 (20.5)		30 (15.0)	34 (17.0)	95 (47.5)		129 (64.5)	71 (35.5)		125 (61.9)	77 (38.1)	
	III	21 (10.9)		38 (19.6)	38 (19.8)	95(19.5)		133 (69.3)	59(30.7)		134 (69.4)	59 (30.6)	
	IV	4 (18.2)		4 (18.2)	4 (18.2)	10 (45.5)		14 (63.6)	8 (36.4)		14 (63.6)	8 (36.4)	
Deficient mismatch repair	Absence	76 (17.3)		76 (17.3)	81 (18.5)	206 (46.9)	0.006*	287 (65.4)	152 (34.6)	0.017*	283 (64.2)	158 (35.8)	0.037*
	Presence	14 (35.9)		7 (17.9)	6 (15.4)	12 (30.8)		18 (46.2)	21 (53.8)		19 (47.5)	21 (52.5)	
Neoadjuvant chemoradiotherapy	Absent	88 (18.2)		77 (16.5)	86 (18.5)	215 (46.1)	0.025*	301 (64.6)	165 (35.4)	0.026*	293 (62.5)	176 (37.5)	0.375
	Present	2 (16.7)		6 (50.0)	1 (8.3)	3 (25.0)		4 (33.3)	8 (66.7)		9 (75.0)	3 (25.0)	
Local recurrence	Absence	67 (22.3)		51 (17.0)	52 17.3)	130 (43.3)	0.031*	182 (60.7)	118 (39.3)	0.064	181 (59.9)	121 (40.1)	0.093
	Presence	23 (12.9)		32 (18.0)	35 (19.7)	88 (49.4)		123 (69.1)	55 (30.9)		121 (67.6)	58 (32.4)	
Disease-specific death	Alive	69 (24.0)		49 (17.1)	48 (16.7)	121 (42.2)	0.002*	169 (58.9)	118 (41.1)	0.006*	170 (58.8)	119 (41.2)	0.027*
	Dead	21 (11.0)		34 (17.8)	39 (20.4)	97 (50.8)		136 (71.2)	55 (28.8)		132 (68.8)	60 (31.3)	

Cases without clinical information or for which data are not assessable due to cautery artifact, fragmentation, or incorrect orientation of tumour tissues are excluded from statistical analyses

High CD8 expression is significantly associated with male gender (*p* = 0.026), low T category (*p* = 0.001), and no lymph node metastasis (*p* = 0.009). Notably, Patients with neoadjuvant chemoradiotherapy significantly increase CD8 expression (*p* = 0.026).

Positive PD-L1 expression is also significantly associated with low T category (*p* = 0.001) and no lymph mode metastasis (*p* = 0.002). However, there is no relation to neoadjuvant chemoradiotherapy (*p* = 0.375).

Relationships among biomarkers of the tumour microenvironment are shown in Table 3. Positive PD-L1 expression is significantly associated with dMMR, high CD8 expression, and high PD-1 expression (*p* = 0.037, *p* <  0.001, *p* <  0.001, respectively). High CD8 expression is also detected in tumour samples with high PD-1 expression (*p* <  0.001) and dMMR (*p* = 0.017).

**Table 3:** Relationships among expression of PD-L1, CD8, and PD-1 with deficient mismatch repair

Parameters		PD-L1 expression (%)			CD8 expression (%)			
		Negative	Positive	*P*		Low	High	*P*
CD8 expression	Low	218 (71.5)	87 (28.5)	< 0.001*	–		–	–
	High	83 (48.0)	90 (52.0)		–		–	–
PD-1 expression	Low	214 (70.4)	90 (29.6)	< 0.001*	238 (78.8)		64 (21.2)	< 0.001*
	High	88 (49.7)	89 (50.3)		67 (38.1)		109 (61.9)	
Deficient mismatch repair	Absence	283 (64.2)	158 (35.8)	0.037*	287 (65.4)		152 (34.6)	0.017*
	Presence	19 (47.5)	21 (52.5)		18 (46.2)		21 (53.8)	

Cases without clinical information or with unassessable data due to cautery artifact, fragmentation, or incorrect orientation of tumour tissues are excluded from statistical analyses

### Univariate and multivariate analyses for overall survival

**Fig. 2:**
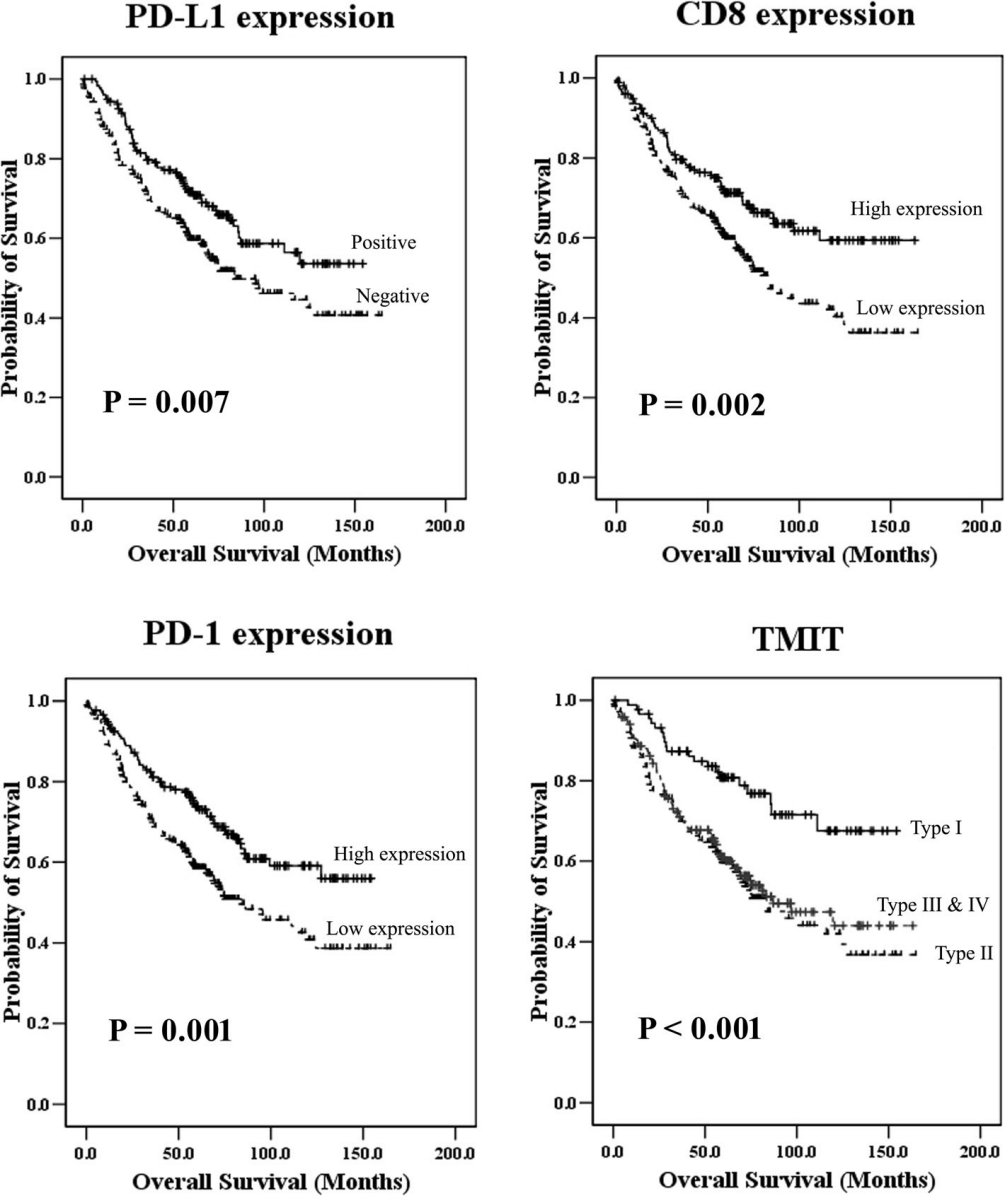
Kaplan-Meier curves of overall survival

In univariate analyses for overall survival (OS) with the log-rank method (Table 4), the dependence of clinical prognosis is significant for TMIT (*p* <  0.001); PD-L1 (*p* = 0.007), CD8 (*p* = 0.002), and PD-1 (*p* = 0.001) expression levels; and several clinicopathological parameters, such as patient age (*p* <  0.001), lymph vascular invasion (*p* <  0.001), perineural invasion (*p* = 0.003), and pTNM stage (*p* <  0.001). The TMIT I subgroup is associated with better OS than other TMIT. The TMIT II subgroup is associated with the worst OS. PD-L1 positivity, high CD8, or high PD-1 expression is significantly associated with favourable OS (Kaplan-Meier curve, Fig. 2). Notably, dMMR (*p* = 0.014) and chemo- or radiotherapy (*p* <  0.001) are generally also associated with favourable clinical outcomes.

**Table 4:** Univariate analysis (Log-rank test) for overall survival

Parameters		Mean Survival (Months)	Confidence Interval (95%)		*P*
			Lower	Upper	
Age	Less than 67 yrs	116.5	107.2	125.8	< 0.001*
	More than 67 yrs	87.6	60.3	85.9	
Sex	Male	103.9	95.7	112.2	0.330
	Female	94.1	83.3	105.0	
Size	Less than 4.9 cm	92.6	84.1	101.0	0.109
	More than 4.9 cm	107.2	97.6	116.8	
Location	Ascending	100.2	84.6	115.7	0.716
	Transverse to sigmoid	98.9	90.3	107.6	
	Rectum	95.8	83.4	108.1	
Histologic type	Intestinal type	101.1	94.4	107.8	0.856
	Mucinous type	82.0	55.6	108.3	
Differentiation	Well	106.4	90.5	122.2	0.117
	Moderately	99.8	92.5	107.0	
	Poorly	88.4	61.1	115.7	
Lymphovascular invasion	Absence	109.8	101.7	117.8	< 0.001*
	Presence	77.3	66.6	88.0	
Perineural invasion	Absence	94.9	87.2	102.6	0.003*
	Presence	68.0	52.1	84.0	
pT category	pT1–2	112.2	99.9	124.4	< 0.001*
	pT3	100.3	92.6	108.1	
	pT4	73.8	54.7	92.8	
Lymph node metastasis	Absence	113.3	104.6	122.0	< 0.001*
	Presence	84.9	75.2	94.6	
Stage	I	100.9	88.4	113.4	< 0.001*
	II	113.8	103.8	123.7	
	III	88.9	78.7	99.1	
	IV	37.2	19.7	54.6	
Deficient mismatch repair	Absence	98.9	92.0	105.8	0.014*
	Presence	126.8	105.8	147.8	
Chemo- or radiotherapy	Absence	83.3	72.1	94.4	< 0.001*
	Presence	109.5	101.7	117.4	
Local recurrence	Absence	147.6	142.1	153.2	< 0.001*
	Presence	42.2	37.4	46.9	
PD-1 expression	Low	93.2	84.5	101.9	0.001*
	High	109.2	99.9	118.5	
CD8 expression	Low	92.4	83.9	101.0	0.002*
	High	114.8	104.4	125.2	
PD-L1 expression	Negative	94.9	86.3	103.4	0.007*
	Positive	107.1	97.7	116.6	
Tumour microenvironmentimmune type	Type I (PD-L1+/CD8H)	122.1	110.2	133.9	< 0.001*
	Type III (PD-L1+/CD8L) & Type IV (PD-L1−/CD8H)	96.9	85.8	108.0	
	Type II (PD-L1−/CD8L)	92.4	82.2	102.6	

Cases with no clinical information or for which the data are not assessable due to cautery artifact, fragmentation, or incorrect orientation of tumour tissues are excluded in statistical analyses

In multivariate survival analyses with the Cox proportional hazard model (Table 5), the TMIT I subgroup has the best survival benefit, and the TMIT II subgroup has the worst survival benefit, while the TMIT III and IV subgroups have intermediate survival benefits. Low CD8 expression is also an independent and unfavourable prognosticator for OS, in addition to higher pTNM stage, more than 67 years in age, lymph vascular invasion, and only surgical resection without chemo- or radiotherapy.

**Table 5:** Multivariate analysis (Cox proportional hazards model)

Parameters		Multivariate (TMIT)			Multivariate (PD-L1/CD8 TIL)		
		HR	95% CI	*P*	HR	95% CI	*P*
TMIT				0.022*			**–**
	Type III & IV vs. Type I	1.831	1.122–2.989	0.015*	–	–	–
	Type II vs. Type I	1.955	1.208–3.164	0.006*	–	–	–
CD8	Low vs. high expression	–	–	**–**	1.406	1.021–1.936	0.037*
PD-L1	Positive vs. Negative expression	–	–	**–**	1.239	0.903–1.700	0.184
pT category				< 0.001*			< 0.001*
	pT3 vs. pT1–2	2.029	1.209–3.406	0.007*	2.072	1.230–3.488	0.006*
	pT4 vs. pT1–2	3.716	2.027–6.813	< 0.001*	3.838	2.092–7.039	< 0.001*
Lymph node metastasis	Presence vs. Absence	1.899	1.408–2.561	< 0.001*	1.955	1.450–2.636	< 0.001*
Deficient mismatch repair	Absence vs. Presence	1.780	0.871–3.640	0.114	1.827	0.894–3.732	0.098
Age	More than 67 vs. less than 67 years	1.687	1.224–2.326	0.001*	1.662	1.207–2.290	0.002*
Chemotherapy or Radiotherapy	Only resection vs. Resection with chemo- or radiotherapy	2.039	1.491–2.789	< 0.001*	2.086	1.526–2.852	< 0.001*

*CI* confidence interval, *HR* hazard ratio, *TIL* tumour-infiltrating lymphocytes, *TMIT* tumour microenvironment immune type

## Discussion

A four-tiered classification for tumour microenvironment immune type (TMIT) has been proposed to describe the patient’s immune status and to determine immunotherapy-responsive subgroups [18]. Each TMIT is defined as follows: Type I, PD-L1 positivity with TIL (adaptive immune resistance); Type II, PD-L1 negativity with no TIL (immune ignorance); Type III, PD-L1 positivity with no TIL (intrinsic induction); and Type IV, PD-L1 negativity with TIL (possible role of other suppressors in producing immune tolerance). In this study, we corroborated the prognostic implications for each TMIT assigned to the colorectal adenocarcinomas according to PD-L1 expression and TIL. Types I and II were associated with the best and worst prognoses, respectively, while Types III and IV had intermediate outcomes in the overall survival analyses.

In colorectal adenocarcinoma, the prognostic value of PD-L1 expression has been contradictory*.* Our results are consistent with those of *Huang* et al. [7], who demonstrated that high PD-L1 expression on tumour cells was associated with improved disease-free survival and overall survival. Some studies [8–10] indicated that PD-L1-positive immunoreactivity on tumour cells was a significant predictor of unfavourable overall, disease-free, or recurrence-free survival in colorectal adenocarcinoma. However, other studies [11, 12] reported that PD-L1 expression in tumour cells was not associated with clinical prognosis, regardless of MSI. Plausible explanations for these contradictory prognostic values for PD-L1 expression are as follows: 1) various methodologies such as different primary antibodies and arbitrary cut-off values for PD-L1 immune expression, 2) tumour heterogeneity, 3) diverse patient populations, and 4) complex interactions of tumour immune microenvironments. To enhance the representativeness and overcome tumour heterogeneity, five non-contiguous microscopic hotspots representing the densest immune or tumour cells were selected. Additionally, we attempted to simplify the complexity of assessing the tumour immune microenvironment using a more concise and representative set of immune biomarkers, such as PD-L1, PD-1, and CD8.

In contrast with contradictory results for PD-L1 expression, CD8 overexpression has been a constantly favourable prognostic factor in many studies [15, 19, 20]. Especially, neoadjuvant chemoradiotherapy enhances CD8 expression as shown in our study.

Pathogenetic analysis for TIL, which are intermingled with tumour cells, plays a crucial role in interpreting tumorigenesis and predicting a clinical biologic outcome. TIL can boost PD-L1 expression in tumour cells in an interferon-gamma (IFN-γ)-dependent manner. PD-L1 overexpression can, in turn, trigger apoptosis and immune tolerance of T-cells [21]. IFN-γ facilitates PD-L1 expression in tumour cells through the JAK-STAT (signal transducer and activator of transcription) pathway [22]. Therefore, CD8-positive TIL in the stroma of colorectal adenocarcinoma is significantly associated with positive PD-L1 expression. Capitalizing on this background and consistent with the positive correlation of PD-1 expression with CD8 and PD-L1 expression as shown in our study, patients with TMIT I tumours can represent a stronger CD8/PD-L1/PD-1 interaction compared to other TMIT subgroups. A more patent CD8/PD-L1/PD-1 concurrence is a strong indicator that immune checkpoint inhibitors such as PD-L1 or PD-1 blockers are more effective for colorectal adenocarcinoma patients in the TMIT I subgroup.

PD-L1 overexpression in colorectal adenocarcinoma is implicated in increased tumour mutation burden, MSI, and upregulated immune-related genes [23–25]. *Ock* et al. [26] reported that the TMIT I subgroup is related to a high mutation burden and PD-L1 amplification. *Madore* et al. [27] reported that PD-L1-positive tumours in stage III melanoma had increased levels of immune-associated genes, suggesting that PD-L1 expression indicates an upregulation of cytotoxic (CD8) T-cell- or macrophage-related genes. Clues gleaned from these recent studies combined with our results suggest that PD-L1 overexpression in colorectal adenocarcinoma is canonically or non-canonically associated with increased antigenic recognition of tumours (anti-tumorigenicity by TIL) through MSI, increased tumour mutation burden or IFN-γ secretion by TIL, although elucidating these pathogenetic mechanisms needs further study.

To date, few studies of colorectal adenocarcinoma have attempted to classify tumour microenvironment complexity with multiple immune markers to identify specific subpopulations of colorectal adenocarcinoma patients for evidence-based, targeted immune therapies. *Chen* et al. [15] reported that both PD-L1 positivity and CD8-high TIL predict favourable clinical outcomes for locally advanced rectal cancer patients treated with neoadjuvant chemoradiotherapy. Notably, *Huang* et al. [7] reported results consistent with our study: the subgroup of patients with high CD8-high TIL and high PD-L1 expression in tumour cells have better survival outcomes than other subgroups. These integrative analyses with multiple biomarkers underscore the importance of evaluating both PD-L1 expression and TIL infiltration to determine the subgroup responsive to immune checkpoint inhibitors.

Collective lines of evidence support that the TMIT I subgroup (PD-L1-positive tumour cells and high CD8-positive TIL) is associated with the following favourable biologic behaviours: 1) high CD8-positive TIL is a favourable prognosticator; 2) PD-L1 expression is closely linked to immune reactivation through an increased tumour mutation burden and upregulated immune-related genes; 3) In colorectal adenocarcinomas, MSI significantly associated with PD-L1 expression in tumour cells have a favourable clinical prognosis; 4) the blocking capacity of immune checkpoint inhibitors is significantly correlated with an increased tumour mutation burden that is indicated by PD-L1 overexpression [14].

In summary, we ascertained the prognostic value of a four-tiered classification of tumour microenvironment immune types (TMIT) in colorectal adenocarcinomas according to PD-L1 expression and TIL status. Type I (both PD-L1-positive tumour cells and high-CD8 TIL) and Type II (PD-L1-negative tumour cells and low-CD8 TIL) are associated with the best and worst prognoses, respectively. Positive PD-L1 expression is significantly associated with dMMR and correlated with high-CD8 or PD-1 overexpression of TIL, which indicates that the TMIT I subgroup represents a stronger CD8/PD-L1/PD-1 interaction than the other TMIT. Therefore, the TMIT I subgroup may be a beneficial candidate to predict a better response rate to immune checkpoint inhibitors by hindering CD8/PD-L1/PD-1 interaction and giving rise to immune reactivation. Based on their tumour microenvironment immune reactions, such categorized immune subgroups of colorectal cancers can be predictive for clinical prognosis. TMIT classification also provides helpful options for determining which subgroups are immunotherapy-targetable to elicit an effective response.

## Conclusion

TMIT I (both PD-L1-positive tumour cells and high-CD8 TIL) is associated with the best prognosis, and shows stronger CD8/PD-L1/PD-1 signalling interaction compared to the other TMIT. Therefore, we propose that the TMIT I subgroup is a candidate TMIT to predict effective response rate for existing immune checkpoint inhibitors and determine targetable subgroups for emerging therapies.

## Data Availability

The datasets used and/or analysed during the current study are available from the corresponding author on reasonable request.

## Abbreviations

CD8: Cluster of differentiation
dMMR: Deficient mismatch repair
FFPE: Formalin-fixed, paraffin-embedded
H&E: Haematoxylin and eosin
IFN-γ: Interferon-gamma
IHC: Immunohistochemical
IRS: Immunoreactive scores
MSI: Microsatellite instability
OS: Overall survival
PD-1: Programmed cell death protein 1
PD-L1: Programmed death-ligand 1
SD: Standard deviation
STAT: Signal transducer and activator of transcription
TIL: Tumour-infiltrating lymphocytes
TMA: Tissue microarray
TMB: Tumour mutational burden
TMIT: Tumour microenvironment immune type

## References

[1] Arnold M, Sierra MS, Laversanne M, Soerjomataram I, Jemal A, Bray F (2017). Global patterns and trends in colorectal cancer incidence and mortality. Gut.

[2] Thota R, Gonzalez RS, Berlin J, Cardin DB, Shi C (2017). Could the PD-1 pathway be a potential target for treating small intestinal adenocarcinoma?. Am J Clin Pathol.

[3] Herbst RS, Soria JC, Kowanetz M, Fine GD, Hamid O, Gordon MS (2014). Predictive correlates of response to the anti-PD-L1 antibody MPDL3280A in cancer patients. Nature..

[4] Taieb J, Moehler M, Boku N, Ajani JA, Yanez Ruiz E, Ryu MH (2018). Evolution of checkpoint inhibitors for the treatment of metastatic gastric cancers: current status and future perspectives. Cancer Treat Rev.

[5] Topalian SL, Hodi FS, Brahmer JR, Gettinger SN, Smith DC, McDermott DF (2012). Safety, activity, and immune correlates of anti-PD-1 antibody in cancer. N Engl J Med.

[6] Yi M, Jiao D, Xu H, Liu Q, Zhao W, Han X (2018). Biomarkers for predicting efficacy of PD-1/PD-L1 inhibitors. Mol Cancer.

[7] Huang CY, Chiang SF, Ke TW, Chen TW, You YS, Chen WT (2018). Clinical significance of programmed death 1 ligand-1 (CD274/PD-L1) and intra-tumoral CD8+ T-cell infiltration in stage II-III colorectal cancer. Sci Rep.

[8] Lee KS, Kim BH, Oh HK, Kim DW, Kang SB, Kim H (2018). Programmed cell death ligand-1 protein expression and CD274/PD-L1 gene amplification in colorectal cancer: implications for prognosis. Cancer Sci.

[9] Enkhbat T, Nishi M, Takasu C, Yoshikawa K, Jun H, Tokunaga T (2018). Programmed cell death ligand 1 expression is an independent prognostic factor in colorectal Cancer. Anticancer Res.

[10] Lee LH, Cavalcanti MS, Segal NH, Hechtman JF, Weiser MR, Smith JJ (2016). Patterns and prognostic relevance of PD-1 and PD-L1 expression in colorectal carcinoma. Mod Pathol.

[11] Eriksen AC, Sorensen FB, Lindebjerg J, Hager H (2019). DePont Christensen R, Kjaer-Frifeldt S, et al. Programmed Death Ligand-1 expression in stage II colon cancer - experiences from a nationwide populationbased cohort. BMC Cancer.

[12] Wang L, Liu Z, Fisher KW, Ren F, Lv J, Davidson DD (2018). Prognostic value of programmed death ligand 1, p53, and Ki-67 in patients with advanced-stage colorectal cancer. Hum Pathol.

[13] Brahmer JR, Tykodi SS, Chow LQ, Hwu WJ, Topalian SL, Hwu P (2012). Safety and activity of anti-PD-L1 antibody in patients with advanced cancer. N Engl J Med.

[14] Yarchoan M, Hopkins A, Jaffee EM (2017). Tumor mutational burden and response rate to PD-1 inhibition. N Engl J Med.

[15] Chen TW, Huang KC, Chiang SF, Chen WT, Ke TW, Chao KSC (2019). Prognostic relevance of programmed cell death-ligand 1 expression and CD8+ TILs in rectal cancer patients before and after neoadjuvant chemoradiotherapy. J Cancer Res Clin Oncol.

[16] Zhao P, Li L, Jiang X, Li Q (2019). Mismatch repair deficiency/microsatellite instability-high as a predictor for anti-PD-1/PD-L1 immunotherapy efficacy. J Hematol Oncol.

[17] Ascierto PA, Agarwala S, Botti G, Cesano A, Ciliberto G, Davies MA (2016). Future perspectives in melanoma research : Meeting report from the "Melanoma Bridge". Napoli, December 1st-4th 2015. J Transl Med.

[18] Teng MW, Ngiow SF, Ribas A, Smyth MJ (2015). Classifying cancers based on T-cell infiltration and PD-L1. Cancer Res.

[19] Governa V, Trella E, Mele V, Tornillo L, Amicarella F, Cremonesi E (2017). The interplay between neutrophils and CD8(+) T cells improves survival in human colorectal Cancer. Clin Cancer Res.

[20] Ledys F, Klopfenstein Q, Truntzer C, Arnould L, Vincent J, Bengrine L (2018). RAS status and neoadjuvant chemotherapy impact CD8+ cells and tumor HLA class I expression in liver metastatic colorectal cancer. J Immunother Cancer.

[21] Chiu YM, Tsai CL, Kao JT, Hsieh CT, Shieh DC, Lee YJ (2018). PD-1 and PD-L1 up-regulation promotes T-cell apoptosis in gastric adenocarcinoma. Anticancer Res.

[22] Mimura K, Teh JL, Okayama H, Shiraishi K, Kua LF, Koh V (2018). PD-L1 expression is mainly regulated by interferon gamma associated with JAK-STAT pathway in gastric cancer. Cancer Sci.

[23] Rosenbaum MW, Bledsoe JR, Morales-Oyarvide V, Huynh TG, Mino-Kenudson M (2016). PD-L1 expression in colorectal cancer is associated with microsatellite instability, BRAF mutation, medullary morphology and cytotoxic tumor-infiltrating lymphocytes. Mod Pathol.

[24] Inaguma S, Lasota J, Wang Z, Felisiak-Golabek A, Ikeda H, Miettinen M (2017). Clinicopathologic profile, immunophenotype, and genotype of CD274 (PD-L1)-positive colorectal carcinomas. Mod Pathol.

[25] Salem ME, Puccini A, Grothey A, Raghavan D, Goldberg RM, Xiu J (2018). Landscape of tumor mutation load, mismatch repair deficiency, and PD-L1 expression in a large patient cohort of gastrointestinal cancers. Mol Cancer Res.

[26] Ock CY, Keam B, Kim S, Lee JS, Kim M, Kim TM (2016). Pan-Cancer Immunogenomic perspective on the tumor microenvironment based on PD-L1 and CD8 T-cell infiltration. Clin Cancer Res.

[27] Madore J, Strbenac D, Vilain R, Menzies AM, Yang JY, Thompson JF (2016). PD-L1 negative status is associated with lower mutation burden, differential expression of immune-related genes, and worse survival in stage III melanoma. Clin Cancer Res.

